# A Lonelier World after COVID-19: Longitudinal Population-Based Study of Well-Being, Emotional and Social Loneliness, and Suicidal Behaviour in Slovenia

**DOI:** 10.3390/medicina60020312

**Published:** 2024-02-12

**Authors:** Vita Poštuvan, Nina Krohne, Meta Lavrič, Vanja Gomboc, Diego De Leo, Lucia Rojs

**Affiliations:** 1 Slovene Centre for Suicide Research, Andrej Marušič Institute, University of Primorska, 6000 Koper, Slovenia; nina.krohne@iam.upr.si (N.K.); meta.lavric@iam.upr.si (M.L.); vanja.gomboc@iam.upr.si (V.G.); diego.deleo@upr.si (D.D.L.); lucia.rojs@iam.upr.si (L.R.); 2 Department of Psychology, Faculty of Mathematics, Natural Sciences and Information Technologies, University of Primorska, 6000 Koper, Slovenia

**Keywords:** COVID-19, loneliness, well-being, suicide

## Abstract

*Background and Objectives*: The impact of coronavirus disease 2019 (COVID-19) goes beyond the consequences of the infectious disease, especially as the measures taken to prevent the spread of the virus have had a very profound impact on people’s social relationships and everyday lives. Several studies have investigated these effects, but there is a lack of longitudinal studies in Central Europe. Objective: The aim of our study was to observe changes in well-being, loneliness, and suicidal behaviour before, during, and after the COVID-19 pandemic using the same population-based cohort. *Materials and Methods:* A representative sample of 444 participants completed online questionnaires at four time points: 2019 (wave 0), 2021 (wave 1), 2022 (wave 2), and 2023 (wave 3). *Results*: The results show significant changes in the levels of well-being and emotional loneliness over these periods. In particular, emotional loneliness increased during the pandemic, followed by a later decrease. Well-being appeared to increase after pandemic-related restrictions diminished but decreased again one year later. No significant changes concerning social loneliness and suicidal ideation were observed. *Conclusions*: Our study suggests that the COVID-19 pandemic changed the way in which people perceive their well-being and especially their relationships with others. From the data, we can conclude that people’s worldview is now lonelier than before the pandemic.

## 1. Introduction

In March 2020, the Slovene government announced a nationwide lockdown with the public strictly instructed to socially distance, self-isolate, and not move outside their own household. The impact of coronavirus disease 2019 (COVID-19), therefore, goes beyond the consequences of the infectious disease, even though the number of infections and deaths associated with this disease is unprecedented. The measures and restrictions to prevent and control COVID-19 have significantly changed lives during the first waves of the disease, and the consequences were observable on social and personal levels, including mental health [1].

The reports of reduced well-being and mental health problems have been published in several studies in different parts of the world. Within just two weeks of the initial outbreak in China, more than half of people (53.8%) reported a moderate to severe impairment of their mental health [2]. A significant percentage (28.8%) showed symptoms of anxiety, while a smaller percentage reported symptoms of depression (16.5%) and increased stress (8.1%). Remarkably, these mental health problems persisted to a similar extent even after one month had passed. An 8.1% increase in experiences of stress was also observed in the UK at the beginning of the pandemic [3]. Italy also reported an increase in the proportion of people struggling with mental health problems. Data from the general population showed that 21.2% of people suffered from depression, 32.6% from anxiety, and 52.4% from insomnia [4].

Studies in Slovenia indicate that, at the beginning of the pandemic, people were concerned about the possible infection of their relatives, especially those who belonged to high-risk groups. These fears were later replaced by concerns about the long-term effects of the pandemic, changes in living conditions, job security, and the economy [5,6]. Excessive worries about one’s own health, the well-being of loved ones, and the future contribute significantly to depressive feelings and a decline in well-being, which was observed in Slovenian samples [7]. Persistent preoccupation with these worries can develop into serious mental health problems, including anxiety disorders, obsessive compulsive disorder, post-traumatic stress disorder, and other related conditions [8].

An important mental health outcome associated with pandemic-related measures of self-isolation and social distancing is loneliness. Killgore et al. [9] showed that, in the USA, the level of loneliness increased significantly in the first three months of the pandemic. In contrast, Luchetti and colleagues [10], who also studied a USA sample, found no significant changes in the first few months of the pandemic. In the UK, Carollo and colleagues [11] observed a U-shaped pattern indicating higher levels of loneliness in the first weeks of lockdown, followed by a decrease in loneliness in the weeks 4–6, and a subsequent increase in loneliness in the following weeks. Studying two different Israeli samples, Einav & Margalit [12] found an increase in loneliness after the COVID-19 pandemic. In Slovenia, Gomboc and colleagues [7] used a similar research design to compare two different samples and found an increase in emotional loneliness (related to the quality of relationships) but a decrease in social loneliness (related to the quantity of relationships).

During the pandemic, loneliness proved to be an important risk factor for suicidal behaviours [13]. The issue of suicidality associated with the COVID-19 pandemic has been addressed in several studies. When studying a population-based cohort in Spain, Ayuso-Mateos and colleagues [14] found no significant differences in suicidal ideation before and during the pandemic. In contrast, Papadopoulou and colleagues [15] found an increase in suicidal ideation in the last two weeks of lockdown compared to the first weeks in a study of the Greek population. This finding was supported by Farooq et al. [13], who conducted a meta-analysis showing that the average prevalence of suicidal ideation reported during the pandemic (12.1%) was higher than the prevalence reported in pre-pandemic studies. However, they argue that the studies predominantly did not use validated measures to assess suicidal ideation. In addition, an increase in suicide-related deaths was observed in some countries [4] but not in the majority [16].

There is clear evidence that mental health deteriorated more in the early stages of the pandemic [17,18,19]. Some studies indicate a gradual decline of the negative impact on mental health as the pandemic progressed [18], suggesting adaptation to the situation [20]. However, studies on mental health in the later phases of the pandemic are sparse and show rather heterogeneous results that vary greatly in different cultural contexts [19,20,21]. In addition, findings on pandemic-related mental health are geographically limited to the English-speaking world as well as some other countries, including the Netherlands, Germany, Italy, China, Japan, Argentina, etc. [17,19]. Little is known about pandemic-related mental health trajectories in regions of Eastern and Central Europe, including Slovenia.

Most studies examining mental health during the pandemic focused predominantly on the period during the pandemic and used a cross-sectional research design [19,20]. By using cross-sectional studies, no causal relationship can be established, so the role of pandemic-related measures on the mental health of the population cannot be assessed. The latter was further often complicated by the use of convenience samples [19]. Studies adopting a longitudinal research design mostly launched as the pandemic began, assessing the trajectory concerning the time of the lockdown, therefore lacking the assessment of long-term mental health consequences as well as meaningful comparisons with people’s mental health status before the pandemic [10,19,20,22].

To overcome the abovementioned shortcomings and address the scarcity of studies concerning the pandemic-related mental health status in Central Europe, we aimed to observe the changes in well-being, loneliness, and suicidal behaviour following the same population-based cohort before, during, and after the COVID-19 pandemic in Slovenia. 

## 2. Materials and Methods

### 2.1. Study Design and Procedure

The data were drawn from a larger dataset of two longitudinal studies, in particular (1) Mental Health Literacy, Destigmatisation of Mental Illnesses and Help-Seeking Behaviour in Times of Distress in Slovenian Adult Population (Slovenian Research and Innovation Agency, Ljubljana, Slovenia [year 2019]) and (2) Individual in the Grip of COVID-19: Psychological Consequences of the Epidemic and Protective Measures to Contain the Spread of Infection (Slovenian Research and Innovation Agency, Ljubljana, Slovenia [years 2021–2023]). The two projects included the same cohort of participants (see Section 2.2.) but focused on different aspects of well-being, mental health, mental health literacy, and other related constructs. However, they shared data on well-being, loneliness, and suicidal behaviour collected before, during, and after the COVID-19 pandemic. These were compared in our study.

The data were collected via an online panel administered by a private company called Valicon (Ljubljana, Slovenia). The company is experienced in conducting research surveys and public polls. It maintains an extensive database of panel members stratified by gender, age, and geographic region. It uses quota sampling so that the allocation of participants within each stratum is a proportional representation of the demographic composition of the total population of Slovenia. 

**Figure 1:**
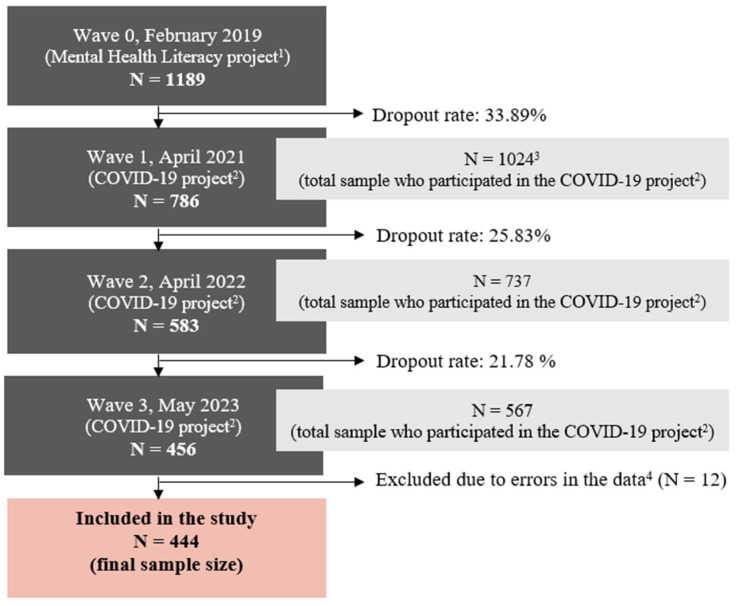
The flowchart of the sampling procedure. The timeline of the study and the sample size for each wave are presented in dark grey boxes on the left side of the flowchart. ^1^ Full name of the project: Mental Health Literacy, Destigmatisation of Mental Illnesses and Help-Seeking Behaviour in Times of Distress in Slovenian Adult Population. ^2^ Full name of the project: Individual in the Grip of COVID-19: Psychological Consequences of the Epidemic and Protective Measures to Contain the Spread of Infection. ^3^ The research design involved the inclusion of new participants from wave 0 to wave 1: 238 new participants joined the study, increasing the sample size presented in the light grey boxes on the right side of the flowchart. The new participants were not included in the analysis. ^4^ The errors included inconsistent entries of certain demographic variables at different measurement waves, disabling meaningful descriptions of the sample.

The procedure comprised several steps, which are described below and illustrated in Figure 1. (i) In wave 0, Valicon sent the invitation to participate in the study by e-mail to the panel members. Panel members who responded to the invitation and gave their informed consent to voluntarily participate in the study were able to complete the survey. Once the predetermined quota for each stratum was reached, no further responses were possible. (ii) In wave 1, the panel members who had responded to the 2019 survey were invited to participate in the study. In order to increase statistical power, additional stratified panel members were invited to participate in the study but were not included in this study. After responding to the e-mail invitation and giving their informed consent, participants completed the survey. (iii) The same cohort was invited to participate in waves 2 and 3. No other panel members were invited to participate in the study for these measurement waves. For the present study, only participants who took part in all four measurement waves were included in the sample (see Section 2.2).

Participants who took part in the study received a participation reward in the form of shopping points. They were rewarded for their engagement in each wave of the survey. Participation was voluntary and only possible upon providing informed consent for study participation. The data were pooled without personally identifying the participants.

This study was conducted in accordance with the Declaration of Helsinki and approved by the Commission of the University of Primorska for Ethics in Human Subjects Research (KER UP) and by the Commission for research ethics in the Department of Psychology at the University of Primorska (details are provided in the Section on Ethics). To mitigate potential risks in exploring difficult topics related to the content of the studies, we provided our contact details and support resources during each wave to inform participants of what support was available in the event of an emergency. Valicon’s legal department advised us on how to ensure the responsible handling of study data in each wave and the consolidation of data across all time points.

### 2.2. Participants

A stratified sample of the general population of Slovenia was included in this study (see Section 2.1). The inclusion criteria were the following: age of the adult at wave 0 (18+ years), proficiency in the Slovenian language, and living in Slovenia at the time of COVID-19 outbreak (waves 1–3). Apart from the quota corresponding to each stratum (see Section 2.1), we did not specify any additional exclusion criteria. The characteristics of the sample are listed in Table 1. 

A total of 1189 participants took part in the study at the baseline (wave 0). Subsequent waves (1–3) were marked by a level of dropout (see Figure 1), leading to the final number of 444 participants. The flowchart of the sample procedure is illustrated in Figure 1. The total dropout rate from wave 0 to wave 3 was 62.66%. To highlight, for longitudinal comparisons, only the data from participants who completed the questionnaires at all measurement waves were used.

Considering the whole sample, gender distribution did not change during this study. There were 211 (47.52%) female and 233 (52.48%) male participants. The age characteristics changed during the years, as the sample aged. These data are presented in Table 1.

### 2.3. Measures

Self-administered online questionnaires were used for the study. In addition to basic socio-demographic data, three questionnaires were used. 

The Paykel Suicide Scale (PSS) [23] contains four items assessing the presence of suicidal thoughts, ideations, and plans in the last two weeks. This is assessed on a six-point scale (from 0 “never” to 5 “always”).

The De Jong Gierveld Scale [24] is a reliable and valid measurement with six items that assess general loneliness and its two aspects: emotional and social loneliness. The items are answered on a three-point scale, indicating whether they agree with the item, disagree, or are indecisive. In our study, we focused on emotional and social loneliness rather than on the general aspect as we were interested in the specifics of people’s perceptions.

The World Health Organization Well-Being Index (WHO–5) [25] measures one’s psychological well-being over the past two weeks through five items using the six-point Likert scale (from 0 “never” to 5 “all the time”).

### 2.4. Statistical Analysis

Frequency analysis and descriptive statistics were used to describe the characteristics of the sample for each wave. The significant difference among the mean values of the outcome variables in four waves were calculated using repeated measures ANOVA. We used a repeated contrast that compares each wave with the previous one and is recommended when the independent variables have a meaningful order [26]—in our case, time. A Greenhouse–Geisser estimate was used in cases of violations in the assumption of sphericity [26]. Additionally, pairwise comparisons between social and emotional loneliness within each wave were performed using Bonferroni-adjusted post hoc tests. Multivariate tests were used to evaluate effect sizes (partial η^2^) for these comparisons. All the tests were two-tailed, with a significance wave of *p* < 0.05. Statistical analysis was performed with R (version 4.1.2) and IBM SPSS (version 25).

## 3. Results

**Figure 2:**
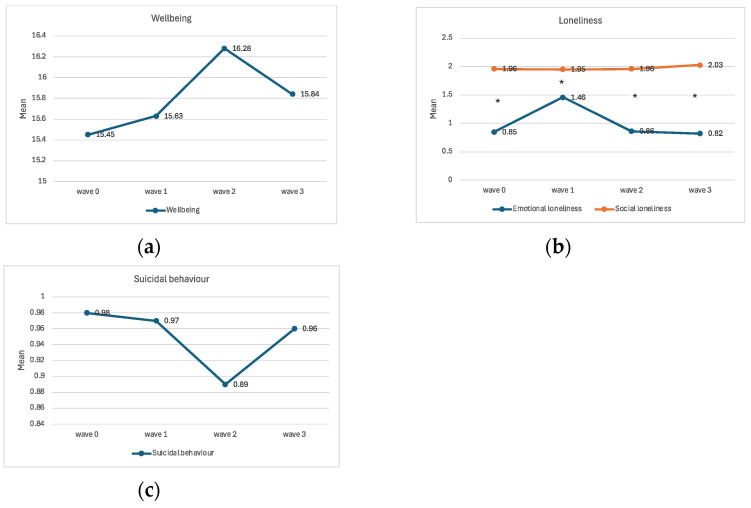
Mean values for (**a**) well-being, (**b**) loneliness (social and emotional), and (**c**) suicidal behaviour during the four waves of this study. * Statistically significant differences of means (*p* < 0.01).

In the present study, four outcome variables (well-being, social loneliness, emotional loneliness, and suicidal behaviour) were examined at four points in time: once before the COVID-19 pandemic (wave 0), once during the pandemic (wave 1), and twice (waves 2 and 3) afterwards. The mean values of these are shown in Figure 2.

The data were analysed using two demographic variables: gender and age. The descriptive statistics of these are summarised in Table 2 and Table 3.

Further analyses were conducted to determine whether the outcome variables changed significantly during the four measurement waves. The results are shown in Table 4. The post hoc comparisons were further calculated to observe the changes in detail (Table 5).

The changes in well-being and emotional loneliness were statistically significantly different in the four waves, while social loneliness and suicidal behaviour showed no statistical differences. In the detailed results, we can see that well-being increased from wave 1 to 2 and decreased from wave 2 to 3, but the effect size is small. Emotional loneliness, however, increased significantly from wave 0 to 1 and decreased from wave 1 to 2, with a large effect size for both changes. Later, from wave 2 to 3, no changes in loneliness were observed.

Across all measurement points from 2019 to 2023, participants consistently reported significantly higher social loneliness than emotional loneliness, with all pairwise comparisons yielding *p* < 0.001. Multivariate analyses indicated strong effect sizes (partial η^2^ ranging from 0.397 to 0.422) for 2019, 2022, and 2023, and a moderate effect size (partial η^2^ = 0.111) for 2021.

## 4. Discussion

We examined the changes before, during, and after the COVID-19 pandemic for the four outcome variables: well-being, social loneliness, emotional loneliness, and suicidal behaviour. 

Our results show that people reported higher levels of social loneliness than emotional loneliness before, during and after the pandemic. Insufficient or unsatisfying social networks appeared to be more common than a perceived lack of intimate connection with other people, which is consistent with previous research [5].

Moreover, our results show a significant increase in emotional loneliness from the period when the COVID-19 pandemic began until 2021. Similar initial increases in loneliness are also documented in other studies [7,11,12,22] and can be understood in the context of the measures taken to prevent the spread of COVID-19 at that time, which directly impacted social relationships. Even though technology tried to compensate for the lack of personal social contacts, interactions via technology did not protect against feelings of loneliness [27,28]. 

When observing the difference between the two components of loneliness, our results show a larger instability in emotional loneliness compared to social loneliness. The results are surprising—an increase in social loneliness, which refers to a perceived lack of social interactions, would be generally expected as a consequence of the COVID-19-related measures of social isolation, while the sharp increase in emotional loneliness was not expected. Emotional loneliness is associated with the feeling of lacking an intimate connection in one’s closest relationships, which should not be severely impacted by preventive measures. During lockdown, people were confined to their homes, which, in Slovenia, mostly consist of other family members, including partners [29]. The results could, therefore, indicate a possible dissatisfaction in people’s intimate partnerships. As Stanley and Markman [30] argue, stress caused by the pandemic can lead to conflicts in one’s partnership. The extended time spent together during the pandemic may have brought to light certain aspects of intimate relationships that had remained hidden in the routine of daily duties before the pandemic. Not only that, but some countries also reported an increase in intimate partner violence during the pandemic [31]. It might also be that other social factors (further) contributed to these increased feelings of emotional loneliness.

Furthermore, the data on the well-being scale do not show any direct changes during the pandemic period, but indicate a mild increase after the end of the pandemic. It seems that overcoming the pandemic may have inspired some optimism, but the decline in the last wave (year 2023) brought the average scores back, closer to the pre-pandemic period. It could also be that, due to the gap in our data in the 2020 assessment period, we have not been able to capture the acute decline in well-being found in other longitudinal studies. This is because these studies reported an increase in mental health problems in the initial phase of the pandemic, which decreased again in the following months. This was understood as an acute and normal reaction to an unforeseen and stressful event, followed by a phase of psychological adaptation and resilience [18,32].

The observed increase in emotional loneliness in 2022 and the decline in well-being in 2023 could also be understood as part of the general decline in mental health documented in several studies focusing on the pandemic period [32], which was exacerbated by the recent crises in Slovenia and Europe. These include economic inflation, armed conflicts, and climate changes with harmful consequences (e.g., fires and floods).

Suicidal behaviour showed no statistically significant changes before, during, and after the pandemic in our study. This is consistent with the findings of Ayuso-Mateos and colleagues [14], who used a similar longitudinal research design for the Spanish population. The data on suicide rates (ratio of people dying by suicide per 100.000) during the pandemic indicate a slight decrease in deaths by suicide in the first year [16]. For Slovenia, the statistical data show this pattern in 2020 [29], with a slight increase thereafter. In our study, however, we focused on suicidal ideation, which is difficult to assess reliably and is considered a rather poor predictor of deaths by suicide [33]. Suicidal behaviours are rare in the general population, and the nuances in change are more difficult to detect compared to studies that focus on a more specific risk group [34].

Our findings, demonstrating continually high levels of social loneliness and dependence of emotional loneliness on external factors (lockdown), recognise loneliness as a key mental health challenge in the post-pandemic era. Considering the well-researched adverse implications of loneliness on mental and physical health [35], we emphasise the need to increase loneliness-related literacy in healthcare providers to recognise and promote social interaction as one of the key pillars of a healthy lifestyle. We recommend the introduction of new or better implementation of existing measures that promote social interaction and social cohesion and support intimate relationships. Ongoing measures to support well-being, including mental health programs and resilience-building initiatives, are critical to addressing post-pandemic challenges. Given the difficulty of predicting suicidal behaviour, we recommend continued vigilance and support for individuals with suicidal thoughts and history of suicide attempt(s). We emphasize the necessity of continuous psychoeducation on suicide prevention not only in healthcare settings but also in schools and working organizations. Given the far-reaching societal impact of recent crises, such as economic inflation and climate change, comprehensive and coordinated action is needed to address mental health challenges.

The present study has several limitations, posing certain constraints on generalizability. Quota sampling focused on gender, age, and region may overlook other important variables (e.g., education, income …) that could influence the research findings, resulting in an under-representation of other relevant demographic or contextual factors. Even if the sample may be representative within each age group, generalizability to the population as a whole may still be limited. Moreover, only the initial sample (collected at wave 0) was sampled to model the general population. However, due to a high dropout rate of 63%, the representativeness of the final sample might have been compromised. An additional factor obstructing generalizability is the panel-based nature of this study. Namely, only panel members were allowed to participate in this study, which might induce a certain level of sampling bias. The latter might be increased by only allowing online participation, although, in Slovenia, 89% of the population regularly uses the Internet [36]. Despite the listed limitations, no other method available in Slovenia could ensure better quality and representativeness of the sample.

Future research directions must focus on the continuous monitoring of the data studied and provide long-term explanations for them. In addition, qualitative studies need to be conducted to provide a deeper understanding of the changes studied and how people perceive them in their daily lives.

## 5. Conclusions

This study aimed to observe the changes in mental health outcomes before, during, and after the pandemic in Slovenia. Specifically, we focused on exploring well-being, loneliness, and suicidal behaviour by following the same cohort of the general population across four time points. With the longitudinal design of this study, our findings allow assessing the impact of COVID-19-related circumstances. By examining the Slovenian population, we contribute a valuable insight into the mental health of an underreached geographical location. 

Our study highlights the lasting impact of the COVID-19 pandemic on social and emotional loneliness. While social loneliness remained persistently high before, during, and after the pandemic, emotional loneliness proved to be far more reactive to external disturbances. This volatility suggests that emotional loneliness is not just an individual experience but a broader societal issue, vulnerable to crises and policy decisions. Addressing long-term social loneliness while mitigating the impact of future disruptions on emotional loneliness should be a key priority for prevention and intervention strategies.

## Figures and Tables

**Table 1:** Sample demographic characteristic in different waves (N = 444).

		Wave 0	Wave 1	Wave 2	Wave 3
Age	18–24 [N (%)]	19 (4.28)	8 (1.80)	6 (1.35)	2 (0.45)
	25–64 [N (%)]	337 (75.90)	317 (71.40)	311 (70.05)	311 (70.05)
	65–79 [N (%)]	87 (19.60)	112 (25.22)	114 (25.67)	117 (26.35)
	>80 [N (%)]	1 (0.22)	7 (1.58)	13 (2.93)	14 (3.15)

**Table 2:** Descriptive data with regard to gender.

Measures		Wave 0	Wave 1	Wave 2	Wave 3
Well-being	All [M ± SD]	15.45 ± 4.67	15.63 ± 5.31	16.28 ± 4.92	15.84 ± 5.11
	Female [M ± SD]	14.80 ± 4.80	14.73 ± 5.39	15.25 ± 5.16	14.94 ± 5.43
	Male [M ± SD]	16.04 ± 4.48	16.45 ± 5.12	17.21 ± 4.51	16.67 ± 4.66
Social Loneliness	All [M ± SD]	1.99 ± 1.18	1.95 ± 1.25	1.96 ± 1.23	2.03 ± 1.22
	Female [M ± SD]	1.82 ± 1.25	1.85 ± 1.28	1.88 ± 1.27	1.89 ± 1.28
	Male [M ± SD]	2.14 ± 1.08	2.03 ± 1.21	2.03 ± 1.20	2.16 ± 1.16
Emotional Loneliness	All [M ± SD]	0.85 ± 1.05	1.46 ± 0.95	0.86 ± 1.05	0.82 ± 1.09
	Female [M ± SD]	0.82 ± 1.05	1.52 ± 0.92	0.91 ± 1.09	0.89 ± 1.13
	Male [M ± SD]	0.89 ± 1.06	1.40 ± 0.97	0.81 ± 1.00	0.76 ± 1.05
Suicidal Behaviour	All [M ± SD]	0.98 ± 2.24	0.97 ± 2.46	0.89 ± 2.28	0.96 ± 2.34
	Female [M ± SD]	1.13 ± 2.52	1.18 ± 2.70	0.94 ± 2.30	0.99 ± 2.26
	Male [M ± SD]	0.84 ± 1.96	0.79 ± 2.21	0.84 ± 2.26	0.94 ± 2.41

**Table 3:** Descriptive data with regard to age groups.

Measures		Wave 0	Wave 1	Wave 2	Wave 3
Well-being	All [M ± SD]	15.45 ± 4.67	15.63 ± 5.31	16.28 ± 4.92	15.84 ± 5.11
	18–24 [M ± SD]	14.95 ± 4.39	12.88 ± 4.85	14.83 ± 3.19	16.0 ± 1.41
	25–64 [M ± SD]	15.05 ± 4.81	15.37 ± 5.28	15.97 ± 4.98	15.39 ± 5.26
	65–79 [M ± SD]	17.06 ± 3.78	16.70 ± 5.20	17.04 ± 4.71	16.97 ± 4.59
	>80 [M ± SD]	/	13.71 ± 6.87	17.54 ± 5.62	16.50 ± 4.97
Social Loneliness	All [M ± SD]	1.99 ± 1.18	1.95 ± 1.25	1.96 ± 1.23	2.03 ± 1.22
	18–24 [M ± SD]	1.95 ± 1.31	2.25 ± 1.17	2.67 ± 0.52	3.00 ± 0.00
	25–64 [M ± SD]	2.04 ± 1.14	1.97 ± 1.23	1.96 ± 1.23	2.06 ± 1.22
	65–79 [M ± SD]	1.82 ± 1.27	1.87 ± 1.28	1.92 ± 1.27	2.02 ± 1.23
	>80 [M ± SD]	/	1.86 ± 1.46	1.92 ± 1.26	1.43 ± 1.28
Emotional Loneliness	All [M ± SD]	0.85 ± 1.05	1.46 ± 0.95	0.86 ± 1.05	0.82 ± 1.09
	18–24 [M ± SD]	1.32 ± 1.20	1.75 ± 1.16	1.17 ± 1.33	1.00 ± 1.41
	25–64 [M ± SD]	0.86 ± 1.06	1.38 ± 0.96	0.85 ± 1.06	0.86 ± 1.13
	65–79 [M ± SD]	0.75 ± 0.99	1.59 ± 0.89	0.81 ± 0.99	0.72 ± 1.02
	>80 [M ± SD]	/	2.43 ± 0.79	1.38 ± 1.12	0.86 ± 1.10
Suicidal Behaviour	All [M ± SD]	0.98 ± 2.25	0.97 ± 2.46	0.89 ± 2.28	0.96 ± 2.34
	18–24 [M ± SD]	1.68 ± 3.53	0.75 ± 0.89	1.33 ± 2.34	0.00 ± 0.00
	25–64 [M ± SD]	0.97 ± 2.24	1.05 ± 2.63	0.93 ± 2.24	1.05 ± 2.48
	65–79 [M ± SD]	0.86 ± 1.88	0.63 ± 1.53	0.54 ± 1.36	0.62 ± 1.43
	>80 [M ± SD]	/	3.14 ± 5.43	2.69 ± 6.07	1.93 ± 4.36

**Table 4:** Differences in time before, during, and after COVID-19.

Measures	F	df	*p*	η^2^
Well-being	5.71	2.87	<0.01	0.01
Social loneliness	0.81	3	0.49	<0.01
Emotional loneliness	72.64	2.88	<0.01	0.14
Suicidal behaviour	0.27	2.77	0.83	<0.01

**Table 5:** Post hoc tests of within-subjects comparisons.

Measures	Comparisons	F	df	*p*	η^2^
Well-being	Wave 0 vs. Wave 1	0.61	1	0.44	<0.01
	Wave 1 vs. Wave 2	10.08	1	<0.01	0.02
	Wave 2 vs. Wave 3	5.70	1	0.02	0.01
Social loneliness	Wave 0 vs. Wave 1	0.44	1	0.51	<0.01
	Wave 1 vs. Wave 2	0.05	1	0.82	<0.01
	Wave 2 vs. Wave 3	1.51	1	0.22	<0.01
Emotional loneliness	Wave 0 vs. Wave 1	125.13	1	<0.01	0.22
	Wave 1 vs. Wave 2	160.40	1	<0.01	0.27
	Wave 2 vs. Wave 3	0.71	1	0.40	<0.01
Suicidal behaviour	Wave 0 vs. Wave 1	<0.01	1	0.97	<0.01
	Wave 1 vs. Wave 2	0.67	1	0.41	<0.01
	Wave 2 vs. Wave 3	0.58	1	0.45	<0.01

## Data Availability

The data presented in this study are available on request from the corresponding author. The data are not publicly available due to due to ongoing research process.
