# Correction: Poštuvan et al. A Lonelier World after COVID-19: Longitudinal Population-Based Study of Well-Being, Emotional and Social Loneliness, and Suicidal Behaviour in Slovenia. *Medicina* 2024, *60*, 312

**DOI:** 10.3390/medicina61101867

**Published:** 2025-10-17

**Authors:** Vita Poštuvan, Nina Krohne, Meta Lavrič, Vanja Gomboc, Diego De Leo, Lucia Rojs

**Affiliations:** 1 Slovene Centre for Suicide Research, Andrej Marušič Institute, University of Primorska, 6000 Koper, Slovenia; nina.krohne@iam.upr.si (N.K.); meta.lavric@iam.upr.si (M.L.); vanja.gomboc@iam.upr.si (V.G.); diego.deleo@upr.si (D.D.L.); lucia.rojs@iam.upr.si (L.R.); 2 Department of Psychology, Faculty of Mathematics, Natural Sciences and Information Technologies, University of Primorska, 6000 Koper, Slovenia

**Error in Figures**

In the original publication [1], there were some mistakes in Figures 1 and 2 as published. In Figure 1, final sample size and number of excluded participants were corrected. The correct Figure 1 appears below.

In Figure 2, there was statistical mistake published. The corrected Figure 2 appears below.

**Figure 2:**
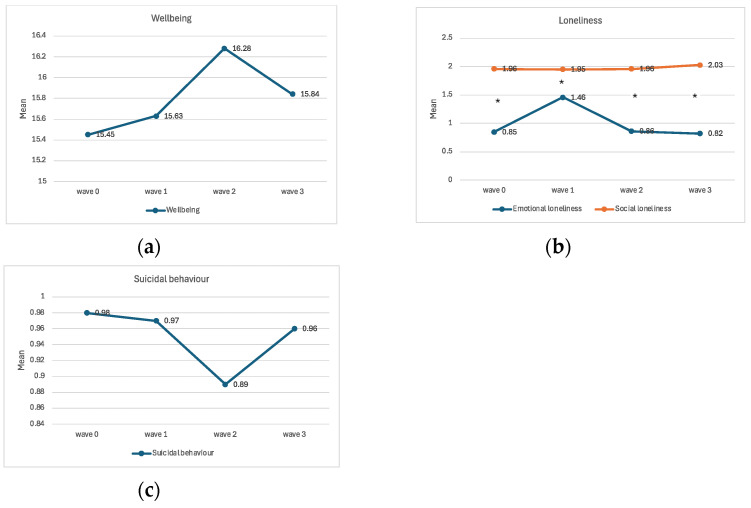
Mean values for (**a**) well-being, (**b**) loneliness (social and emotional), and (**c**) suicidal behaviour during the four waves of this study. * Statistically significant differences of means (*p* < 0.01).

**Error in Tables**

There was an error in the original publication due to the mistake in the number of participants included and excluded from the study, which caused statistical mistakes, so changes were made in Tables 1–5. The corrected tables are presented below.

**Table 1:** Sample demographic characteristic in different waves (N = 444).

		Wave 0	Wave 1	Wave 2	Wave 3
Age	18–24 [N (%)]	19 (4.28)	8 (1.80)	6 (1.35)	2 (0.45)
	25–64 [N (%)]	337 (75.90)	317 (71.40)	311 (70.05)	311 (70.05)
	65–79 [N (%)]	87 (19.60)	112 (25.22)	114 (25.67)	117 (26.35)
	>80 [N (%)]	1 (0.22)	7 (1.58)	13 (2.93)	14 (3.15)

**Table 2:** Descriptive data with regard to gender.

Measures		Wave 0	Wave 1	Wave 2	Wave 3
Well-being	All [M ± SD]	15.45 ± 4.67	15.63 ± 5.31	16.28 ± 4.92	15.84 ± 5.11
	Female [M ± SD]	14.80 ± 4.80	14.73 ± 5.39	15.25 ± 5.16	14.94 ± 5.43
	Male [M ± SD]	16.04 ± 4.48	16.45 ± 5.12	17.21 ± 4.51	16.67 ± 4.66
Social Loneliness	All [M ± SD]	1.99 ± 1.18	1.95 ± 1.25	1.96 ± 1.23	2.03 ± 1.22
	Female [M ± SD]	1.82 ± 1.25	1.85 ± 1.28	1.88 ± 1.27	1.89 ± 1.28
	Male [M ± SD]	2.14 ± 1.08	2.03 ± 1.21	2.03 ± 1.20	2.16 ± 1.16
Emotional Loneliness	All [M ± SD]	0.85 ± 1.05	1.46 ± 0.95	0.86 ± 1.05	0.82 ± 1.09
	Female [M ± SD]	0.82 ± 1.05	1.52 ± 0.92	0.91 ± 1.09	0.89 ± 1.13
	Male [M ± SD]	0.89 ± 1.06	1.40 ± 0.97	0.81 ± 1.00	0.76 ± 1.05
Suicidal Behaviour	All [M ± SD]	0.98 ± 2.24	0.97 ± 2.46	0.89 ± 2.28	0.96 ± 2.34
	Female [M ± SD]	1.13 ± 2.52	1.18 ± 2.70	0.94 ± 2.30	0.99 ± 2.26
	Male [M ± SD]	0.84 ± 1.96	0.79 ± 2.21	0.84 ± 2.26	0.94 ± 2.41

**Table 3:** Descriptive data with regard to age groups.

Measures		Wave 0	Wave 1	Wave 2	Wave 3
Well-being	All [M ± SD]	15.45 ± 4.67	15.63 ± 5.31	16.28 ± 4.92	15.84 ± 5.11
	18–24 [M ± SD]	14.95 ± 4.39	12.88 ± 4.85	14.83 ± 3.19	16.0 ± 1.41
	25–64 [M ± SD]	15.05 ± 4.81	15.37 ± 5.28	15.97 ± 4.98	15.39 ± 5.26
	65–79 [M ± SD]	17.06 ± 3.78	16.70 ± 5.20	17.04 ± 4.71	16.97 ± 4.59
	>80 [M ± SD]	/	13.71 ± 6.87	17.54 ± 5.62	16.50 ± 4.97
Social Loneliness	All [M ± SD]	1.99 ± 1.18	1.95 ± 1.25	1.96 ± 1.23	2.03 ± 1.22
	18–24 [M ± SD]	1.95 ± 1.31	2.25 ± 1.17	2.67 ± 0.52	3.00 ± 0.00
	25–64 [M ± SD]	2.04 ± 1.14	1.97 ± 1.23	1.96 ± 1.23	2.06 ± 1.22
	65–79 [M ± SD]	1.82 ± 1.27	1.87 ± 1.28	1.92 ± 1.27	2.02 ± 1.23
	>80 [M ± SD]	/	1.86 ± 1.46	1.92 ± 1.26	1.43 ± 1.28
Emotional Loneliness	All [M ± SD]	0.85 ± 1.05	1.46 ± 0.95	0.86 ± 1.05	0.82 ± 1.09
	18–24 [M ± SD]	1.32 ± 1.20	1.75 ± 1.16	1.17 ± 1.33	1.00 ± 1.41
	25–64 [M ± SD]	0.86 ± 1.06	1.38 ± 0.96	0.85 ± 1.06	0.86 ± 1.13
	65–79 [M ± SD]	0.75 ± 0.99	1.59 ± 0.89	0.81 ± 0.99	0.72 ± 1.02
	>80 [M ± SD]	/	2.43 ± 0.79	1.38 ± 1.12	0.86 ± 1.10
Suicidal Behaviour	All [M ± SD]	0.98 ± 2.25	0.97 ± 2.46	0.89 ± 2.28	0.96 ± 2.34
	18–24 [M ± SD]	1.68 ± 3.53	0.75 ± 0.89	1.33 ± 2.34	0.00 ± 0.00
	25–64 [M ± SD]	0.97 ± 2.24	1.05 ± 2.63	0.93 ± 2.24	1.05 ± 2.48
	65–79 [M ± SD]	0.86 ± 1.88	0.63 ± 1.53	0.54 ± 1.36	0.62 ± 1.43
	>80 [M ± SD]	/	3.14 ± 5.43	2.69 ± 6.07	1.93 ± 4.36

**Table 4:** Differences in time before, during, and after COVID-19.

Measures	F	df	*p*	η^2^
Well-being	5.71	2.87	<0.01	0.01
Social loneliness	0.81	3	0.49	<0.01
Emotional loneliness	72.64	2.88	<0.01	0.14
Suicidal behaviour	0.27	2.77	0.83	<0.01

**Table 5:** Post hoc tests of within-subjects comparisons.

Measures	Comparisons	F	df	*p*	η^2^
Well-being	Wave 0 vs. Wave 1	0.61	1	0.44	<0.01
	Wave 1 vs. Wave 2	10.08	1	<0.01	0.02
	Wave 2 vs. Wave 3	5.70	1	0.02	0.01
Social loneliness	Wave 0 vs. Wave 1	0.44	1	0.51	<0.01
	Wave 1 vs. Wave 2	0.05	1	0.82	<0.01
	Wave 2 vs. Wave 3	1.51	1	0.22	<0.01
Emotional loneliness	Wave 0 vs. Wave 1	125.13	1	<0.01	0.22
	Wave 1 vs. Wave 2	160.40	1	<0.01	0.27
	Wave 2 vs. Wave 3	0.71	1	0.40	<0.01
Suicidal behaviour	Wave 0 vs. Wave 1	<0.01	1	0.97	<0.01
	Wave 1 vs. Wave 2	0.67	1	0.41	<0.01
	Wave 2 vs. Wave 3	0.58	1	0.45	<0.01

**Text Correction**

There was an error in the original publication due to the mistake in the number of participants. A correction has been made to an Abstract, Section 2.2. Participants, as well as Section 2.4. Statistical Analysis. Changes in the tables resulted in changes in the text after Figure 5 in Section 3. Results, 4. Discussion and Section 5. Conclusions. The corrected paragraphs appear below.

**Abstract:** 

*Materials and Methods:* A representative sample of 444 participants completed online questionnaires at four time points: 2019 (wave 0), 2021 (wave 1), 2022 (wave 2), and 2023 (wave 3). *Results*: The results show significant changes in the levels of well-being and emotional loneliness over these periods. In particular, emotional loneliness increased during the pandemic, followed by a later decrease. Well-being appeared to increase after pandemic-related restrictions diminished but decreased again one year later. No significant changes concerning social loneliness and suicidal ideation were observed.

*2.2. Participants* 

A stratified sample of the general population of Slovenia was included in this study (see Section 2.1).

A total of 1189 participants took part in the study at the baseline (wave 0). Subsequent waves (1–3) were marked by a level of dropout (see Figure 1), leading to the final number of 444 participants. The flowchart of the sample procedure is illustrated in Figure 1. The total dropout rate from wave 0 to wave 3 was 62.66%.

Considering the whole sample, gender distribution did not change during this study. There were 211 (47.52%) female and 233 (52.48%) male participants. The age characteristics changed during the years, as the sample aged. These data are presented in Table 1.

*2.4. Statistical Analysis* 

Additionally, pairwise comparisons between social and emotional loneliness within each wave were performed using Bonferroni-adjusted post hoc tests. Multivariate tests were used to evaluate effect sizes (partial η^2^) for these comparisons.

## 3. Results

The changes in well-being and emotional loneliness were statistically significantly different in the four waves, while social loneliness and suicidal behaviour showed no statistical differences. In the detailed results, we can see that well-being increased from wave 1 to 2 and decreased from wave 2 to 3, but the effect size is small. Emotional loneliness, however, increased significantly from wave 0 to 1 and decreased from wave 1 to 2, with a large effect size for both changes. Later, from wave 2 to 3, no changes in loneliness were observed.

Across all measurement points from 2019 to 2023, participants consistently reported significantly higher social loneliness than emotional loneliness, with all pairwise comparisons yielding *p* < 0.001. Multivariate analyses indicated strong effect sizes (partial η^2^ ranging from 0.397 to 0.422) for 2019, 2022, and 2023, and a moderate effect size (partial η^2^ = 0.111) for 2021.

## 4. Discussion

Our results show that people reported higher levels of social loneliness than emotional loneliness before, during and after the pandemic. Insufficient or unsatisfying social networks appeared to be more common than a perceived lack of intimate connection with other people, which is consistent with previous research [5].

Moreover, our results show a significant increase in emotional loneliness from the period when the COVID-19 pandemic began until 2021.

When observing the difference between the two components of loneliness, our results show a larger instability in emotional loneliness compared to social loneliness. The results are surprising—an increase in social loneliness, which refers to a perceived lack of social interactions, would be generally expected as a consequence of the COVID-19-related measures of social isolation, while the sharp increase in emotional loneliness was not expected. Emotional loneliness is associated with the feeling of lacking an intimate connection in one’s closest relationships, which should not be severely impacted by preventive measures.

Furthermore, the data on the well-being scale do not show any direct changes during the pandemic period, but indicate a mild increase after the end of the pandemic. It seems that overcoming the pandemic may have inspired some optimism, but the decline in the last wave (year 2023) brought the average scores back, closer to the pre-pandemic period.

The observed increase in emotional loneliness in 2022 and the decline in well-being in 2023 could also be understood as part of the general decline in mental health documented in several studies focusing on the pandemic period [32], which was exacerbated by the recent crises in Slovenia and Europe.

Our findings, demonstrating continually high levels of social loneliness and dependence of emotional loneliness on external factors (lockdown), recognise loneliness as a key mental health challenge in the post-pandemic era. Considering the well-researched adverse implications of loneliness on mental and physical health [35], we emphasise the need to increase loneliness-related literacy in healthcare providers to recognise and promote social interaction as one of the key pillars of a healthy lifestyle.

The following sentence was removed from the original manuscript.

“Considering that the levels of social and emotional loneliness remained elevated, we hypothesize that the COVID-19-related measures might have had a longer-lasting effect on how people perceive social interactions or how satisfying intimate relationships are.”

## 5. Conclusions

Our study highlights the lasting impact of the COVID-19 pandemic on social and emotional loneliness. While social loneliness remained persistently high before, during, and after the pandemic, emotional loneliness proved to be far more reactive to external disturbances. This volatility suggests that emotional loneliness is not just an individual experience but a broader societal issue, vulnerable to crises and policy decisions. Addressing long-term social loneliness while mitigating the impact of future disruptions on emotional loneliness should be a key priority for prevention and intervention strategies.

The authors state that the scientific conclusions are unaffected. This correction was approved by the Academic Editor. The original publication has also been updated.

## Figures and Tables

**Figure 1:**
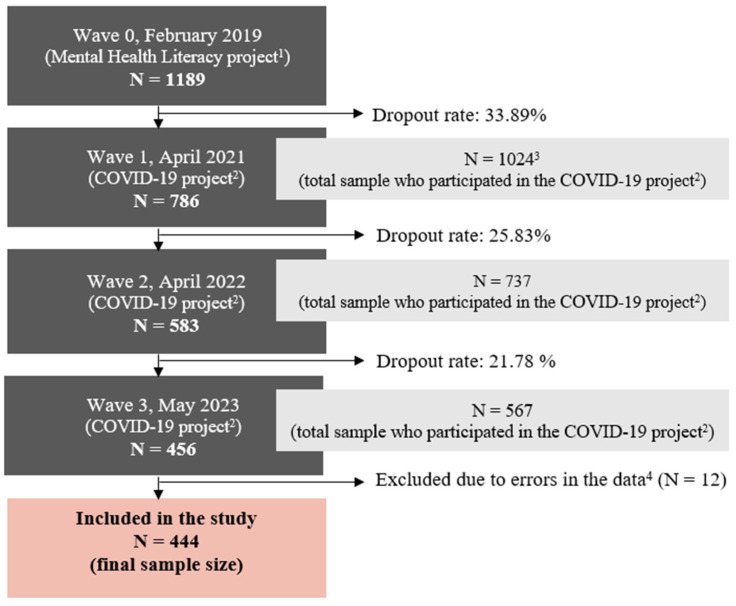
The flowchart of the sampling procedure. The timeline of the study and the sample size for each wave are presented in dark grey boxes on the left side of the flowchart. ^1^ Full name of the project: Mental Health Literacy, Destigmatisation of Mental Illnesses and Help-Seeking Behaviour in Times of Distress in Slovenian Adult Population. ^2^ Full name of the project: Individual in the Grip of COVID-19: Psychological Consequences of the Epidemic and Protective Measures to Contain the Spread of Infection. ^3^ The research design involved the inclusion of new participants from wave 0 to wave 1: 238 new participants joined the study, increasing the sample size presented in the light grey boxes on the right side of the flowchart. The new participants were not included in the analysis. ^4^ The errors included inconsistent entries of certain demographic variables at different measurement waves, disabling meaningful descriptions of the sample.

## References

[1] Poštuvan V., Krohne N., Lavrič M., Gomboc V., De Leo D., Rojs L. (2024). A Lonelier World after COVID-19: Longitudinal Population-Based Study of Well-Being, Emotional and Social Loneliness, and Suicidal Behaviour in Slovenia. Medicina.

